# Integrative transcriptome, proteome, phosphoproteome and genetic mapping reveals new aspects in a fiberless mutant of cotton

**DOI:** 10.1038/srep24485

**Published:** 2016-04-14

**Authors:** Qi-Feng Ma, Chun-Hui Wu, Man Wu, Wen-Feng Pei, Xing-Li Li, Wen-Kui Wang, Jinfa Zhang, Ji-Wen Yu, Shu-Xun Yu

**Affiliations:** 1 State Key Laboratory of Cotton Biology, Institute of Cotton Research of CAAS, Anyang, Henan 455000, China; 2 Department of Plant and Environmental Sciences, New Mexico State University, Las Cruces, NM 88003, USA

## Abstract

To investigate the molecular mechanisms of fiber initiation in cotton (*Gossypium* spp.), an integrated approach combining transcriptome, iTRAQ-based proteome and genetic mapping was taken to compare the ovules of the Xuzhou 142 wild type (WT) with its fuzzless-lintless (*fl*) mutant at −3 and 0 day post-anthesis. A total of 1,953 mRNAs, 187 proteins, and 131 phosphoproteins were differentially expressed (DE) between WT and *fl*, and the levels of transcripts and their encoded proteins and phosphoproteins were highly congruent. A functional analysis suggested that the abundance of proteins were mainly involved in amino sugar, nucleotide sugar and fatty acid metabolism, one carbon pool for folate metabolism and flavonoid biosynthesis. qRT-PCR, Western blotting, and enzymatic assays were performed to confirm the regulation of these transcripts and proteins. A molecular mapping located the lintless gene *li3* in the *fl* mutant on chromosome 26 for the first time. A further in-silico physical mapping of DE genes with sequence variations between *fl* and WT identified one and four candidate genes in the *li3* and *n2* regions, respectively. Taken together, the transcript abundance, phosphorylation status of proteins at the fiber initiation stage and candidate genes have provided insights into regulatory processes underlying cotton fiber initiation.

Upland cotton (*Gossypium hirsutum* L.) is an important cash crop that produces the most important natural resources for the textile industry. Cotton fibers, the longest, fastest-growing cells in plants, are single cells that arise from the outer epidermal layer of the ovule (seed)^1^. Cotton fiber development can be divided into four distinct but overlapping steps, namely, initiation, elongation, secondary cell wall synthesis, and maturation^2^. In *G. hirsutum*, long lint fibers are produced before or on the day of anthesis, i.e., 0 DPA, although only 15–25% of epidermal cells form commercially usable textile lint fibers^3^. In contrast, ovule epidermal cells initiated at and after 3 DPA produce shorter fibers called linters or fuzz that cannot be separated from seeds by ginning.

Fiber mutants that inhibit the growth or development of fiber initials are useful tools for studying molecular events of early fiber development. Previous researchers have characterized many cotton fiber mutants, including lintless, fuzzless, and fuzzless-lintless mutants, and have identified, described, and mapped their quality traits^4^. Two of the best-described cotton fiber mutants are the dominant *N1* and recessive *n2* genes, with both *N1* and *n2* mutants producing naked seeds after ginning, i.e., fuzzless^5^. Two fiber mutants, Ligon lintless-1 (*Li1*) and Ligon lintless-2 (*Li2*) mutants affect lint fiber length^6^. *Li1* is characterized by contorted leaf laminae, twisted branches and limbs, and extremely short fibers of less than 6 mm long, while *Li2* produces a fiber length similar to that of *Li1* but exhibits normal vegetative growth. A third lintless gene, *Lix*, has been described as having plant morphology similar to *Li1* and occurs on chromosome 4, which is homeologous to chromosome 22^7^. The Xuzhou 142 lintless-fuzzless (*fl*) Upland cotton mutant, which possesses no fibers (i.e., no lint and fuzz) unlike the WT, was originally discovered in a commercial Upland cultivar Xuzhou 142^8^. Subsequent studies determined that the mutant is controlled by two pairs of recessive genes with a genotype of *li3li3n2n2*. The *n2* gene was initially assigned to chromosome 26 but was later mapped to chromosome 12, just outside of the *N1* region^9^. However, the chromosome location of *li3* is unknown. The *fl* mutant is in a near-isogenic state with its WT, representing a good model system to study fiber initiation without complications from genetic background differences.

Although the molecular mechanism underlying fiber cell differentiation is poorly understood, increasing evidence suggests that numerous genes, endogenous hormones, and small RNAs may participate in this process. Beasley & Ting showed that auxin and gibberellin accelerate fiber cell growth on *in-vitro* cultured cotton fertilized ovules; and unfertilized ovules require exogenous auxin and gibberellins^10^. After examining 32,789 expressed sequence tags from earlier stages (−3, 0, and 3 DPA) of *G. hirsutum* fibers and ovules, Yang *et al.* observed a notable increase in transcription factors and genes related to hormone metabolism, such as gibberellic acid, ethylene, and brassinosteroid biosynthetic pathways^11^. Taliercio and Boykin also applied spotted cotton oligo-gene microarrays containing 11,000 sequences and found 376 genes up-regulated between 1-DPA fibers and 1-DPA ovules in *G. hirsutum*^12^. Using spotted cotton oligo-gene microarrays containing 1,334 genes, Lee *et al.* identified 30 genes that were noticeably different at 0-DPA ovules of the naked seed (no fuzz) *N1N1* mutant vs. its isogenic line with lint and fuzz, TM-1^13^. In a 2-D electrophoresis and tandem mass spectrometric (MS/MS)-based comparative proteomic analysis of −3-DPA and 0-DPA ovules of Xuzhou 142 *fl* mutant and its WT, Liu *et al.* found 46 differentially expressed proteins (DEPs)^14^. Du *et al.* analyzed differences in proteins of WT cotton (*G. arboreum* L.) and its diploid fuzzless mutant at five developmental time points in ovules (1 to 9 DPA); and they found 71 DEPs, with 45 proteins up-regulated in the WT^15^. Kwak *et al.* studied significantly differentially expressed microRNAs (miRNAs) between Xuzhou 142 and *fl* mutant ovules (1 to 10 DPA). They discovered that 8 miRNAs were up-regulated in the WT, suggesting that miRNAs potentially regulate transcripts in cotton fiber development^16^. Wang *et al.* investigated fiber-initiation-related miRNAs between Xuzhou 142 and *fl* ovules (−3 to 3 DPA) and found 12 up-regulated miRNAs in the WT^17^. These results indicate that cotton fiber differentiation and initiation is a complicated biological process requiring a series of well-orchestrated changes in gene regulation of various physiological and biochemical pathways. However, the authors from above studies were unable to attribute these differentially expressed genes, proteins, and miRNAs to *li3* or *n2* as the two genes were not separated when *fl* and its WT were used.

In our current study, transcriptomic, proteomic, and phosphoproteomic expression profiles were first used in combination to profile *fl* and its WT. The generated omics data reveal some new aspects of metabolic pathways and provide a comprehensive expression reference map for cotton fiber initiation based on the *fl* mutant and its WT. Importantly, *li3* in the *fl* mutant was assigned, similar to *n2*, to chromosome 26 and was mapped within a narrow interval for the first time. A co-localisation of anchoring markers with the predicted genes in the *li3* and *n2* regions allowed the identification of possible candidate genes with differential expression and sequence variation for the two genes. These results provide a foundation to isolate and clone *li3* and *n2* genes and elucidating their functional roles in cotton.

## Results

### Integrated transcriptome, proteome, and phosphoproteome analysis

**Figure 1:**
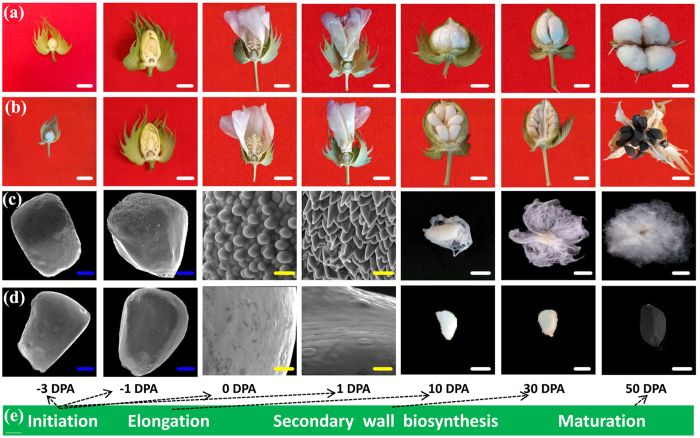
Cotton fiber and boll growth period in WT and *fl*. (**a**,**c**) WT boll and fibre development. (**b**,**d**) *fl* boll development. (**e**) The distinct but overlapping stages include initiation, elongation and secondary wall biosynthesis, and maturation. White bars = 1 cm, Yellow bars = 20 μm, Blue bars = 200 μm.

**Figure 2:**
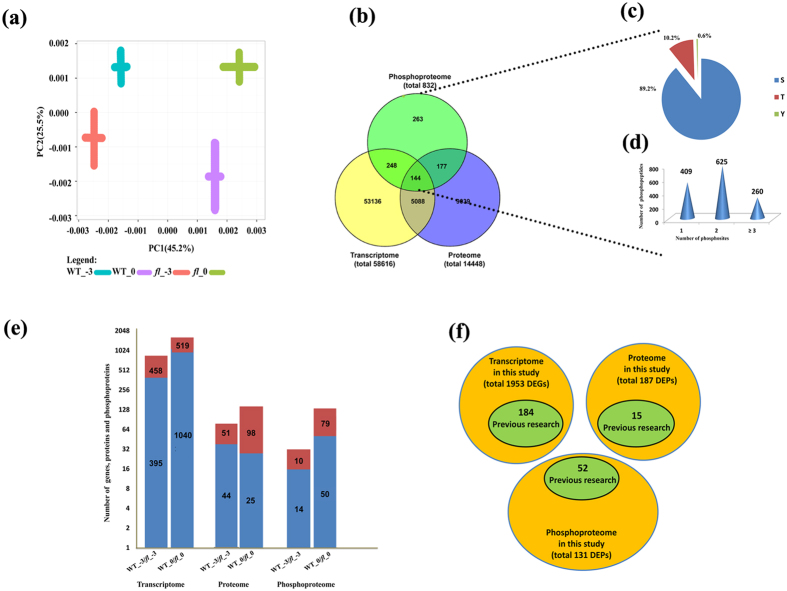
Transcriptome, proteome and phosphoproteome data analysis. (**a**) Principal component analysis among 12 samples. (**b**) Venn diagram of the number of genes/proteins/phosphoproteins in three omics. (**c**) Distribution of the phosphosites. (**d**) Distribution of single and mutilphosphopeptides. (**e**) Distribution of DEGs and DEPs. (**f**) Venn diagram analysis of new DEGs and DEPs.

Previous research and our phenotypic survey revealed that the first detectable sign of fiber initiation occurs on the day of anthesis (i.e., 0 DPA) in WT plants (Fig. 1). In this study, ovules were collected at −3 and 0 DPA to identify differentially expressed genes (DEGs) or differentially expressed proteins/phosphoproteins (DEPs) between WT and its *fl* mutant by RNA-Seq and iTRAQ technology (see Supplementary Fig. S1). To reduce the dimensionality of the data set, the data were transformed by principal component analysis (PCA) into a new set of variables that summarized the data features. The PCA identified the most relevant of 12 samples (i.e., 2 genotypes × 2 stages × 3 replicates), which were divided into four different groups (Fig. 2a).

The 12 libraries included a total of 576 million 90-bp clean reads. A sequencing saturation analysis showed that no less than 48 million clean reads (635-fold of the expression genome coverage) were produced for each sample, adequate for the quantitative analysis of gene expression. By aligning the read sequences to the sequenced upland cotton TM-1 genome containing 76,943 predicted genes, we obtained a total of 58,616 (76%) expressed genes (Fig. 2b).

As a conservative estimate, proteins that shared peptides were placed into the same category, which yielded a total of 5,677 identified protein groups (containing 14,448 proteins). Group leaders, which had the highest number of peptides or corresponded to the longest protein, were assigned to reduce the dimensionality of the data set for each group. Hereafter, the term ‘proteins’ in this paper refers to these group leaders.

To obtain phosphoproteins, phosphorylated peptides were enriched using TiO_2_ before the high-performance liquid chromatography (HPLC)-MS/MS analysis. As a result, 1,294 phosphorylated peptides as well as 2,518 individual phosphorylated sites (phosphosites) originating from 832 phosphoproteins were obtained. Of the 2,518 nonredundant phosphosites, 89.2% were phosphorylated at serine (Ser), 10.2% at threonine (Thr), and 0.6% at tyrosine residues (Fig. 2c). Further study revealed that 409 (31.6%), 625 (48.3%), and 260 (20.1%) of the 1,294 phosphorylated peptides were singly, doubly, and multiply phosphorylated, respectively (Fig. 2d).

A total of 1,953 DEGs were detected using the criteria of false discovery rate (FDR) ≤0.001 and |log2 Ratio| ≥ 1. The number of DEGs between the WT and *fl* during cotton initiation stages was 853 at −3 DPA and 1,559 at 0 DPA. Gene Ontology (GO) annotations and KEGG analyses were performed via Blast2GO software. The KEGG pathway analysis identified various pathways related to metabolic processes, including fatty acid metabolism, one-carbon folate metabolism, and flavonoid biosynthesis. Figure 2e gives an overview of the genes, proteins, and phosphoproteins that were significantly differentially expressed between the WT and *fl* (see Supplementary Table S1). A complete list of genes, proteins, and phosphoproteins is provided in Supplementary Table S2.

Analysis of these diagrams at different omics levels yielded a number of novel findings, including the identification of cotton fiber initiation genes and protein products, few of which have been reported as involved in cotton fiber initiation to date. Of the 1,953 genes, 187 proteins, and 131 phosphoproteins related to fiber initiation identified in this study, only 184, 15, and 52, respectively, were identified in previous research (Fig. 2f and Supplementary Table S3). It is not surprising that our study has uncovered more DEGs and DEPs due to its genomewide coverage from the extremely sequencing depth and better gene annotation using the most recently sequenced tetraploid genome.

### Comparison of gene, protein, and phosphoprotein expression

**Figure 3:**
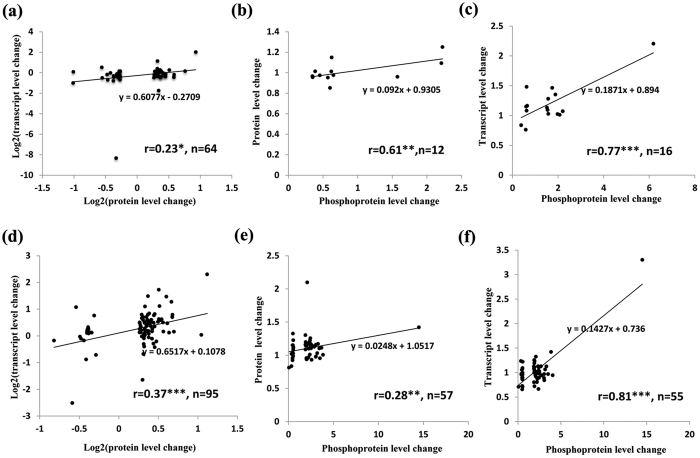
Concordance among changes in the abundance of transcripts, proteins and phosphoproteins. (**a**–**c**) Correlation of transcripts, proteins and phosphoproteins between the WT and *fl* at −3 DPA. (**d**–**f**) Correlation of transcripts, proteins and phosphoproteins between the WT and *fl* at 0 DPA. n is the number of quantified gene products. (Two-tailed test, *p < 0.1, **p < 0.05, ***p < 0.01, p = 0.068 in (**a**), p = 0.031 in (**b**), p = 4.57E-04 in (**c**), p = 2.74E-04 in (**d**), p = 0.03 in (**e**), p = 6.16E-14 in (**f**)).

To compare proteomes, phosphoproteomes, and transcriptomes, we matched DEGs with quantifiable proteins and phosphoproteins. To generate a second list comparing proteins and phosphoproteins with their cognate mRNAs, we rank-ordered the proteins and phosphoproteins in accordance with their expression (WT/*fl*) ratio at −3 or 0 DPA. Positive correlations between transcript and protein, between transcript and phosphoprotein, and between protein and phosphoprotein were observed at both −3 and 0 DPA (Fig. 3). Compared with the correlation between protein and transcript levels, the correlation between phosphoprotein and transcript levels was higher (0.81 vs. 0.37, Fig. 3f) when down-regulated DEP measurements in the WT were considered.

This integrated analysis also uncovered evidence of differential splicing/isoform regulation in the next-generation sequencing data. In our DEG/DEP data, we found that six genes/proteins produced two or more alternative splicing isoforms, one of which was also phosphorylated (see Supplementary Fig. S2). Compared with the *fl* mutant, CotAD_56281 and CotAD_17449 genes in the WT were characterized by alternative 3′ splicing and intron retention, respectively. Conversely, CotAD_25698, CotAD_44282, CotAD_62775 and CotAD_21820 genes featured alternative 3′ splicing and intron retention in *fl*.

Interestingly, we identified one protein, CotAD_44282, that is known to be a crucial protein during fiber development and is involved in negative regulation of heat shock gene expression^18^. One previously unknown phosphosite was identified in this protein at the N-terminus: Ser145, which is conserved between *Medicago truncatula* and *Oryza sativa*, but present in these species as a non-phosphosite. Moreover, this cotton phosphoprotein was the only one containing all four phosphosites simultaneously (see Supplementary Fig. S3).

### Clustering of DEPs to predict novel regulation of fiber initiation

**Figure 4:**
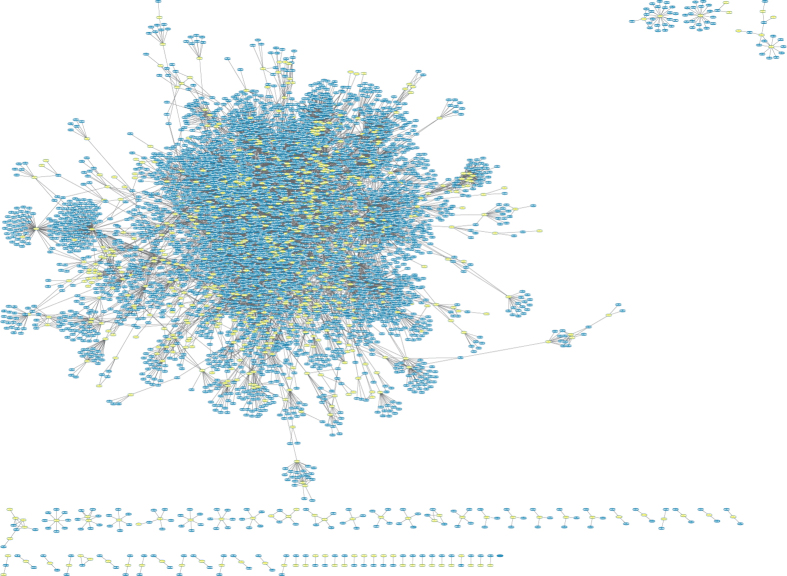
Protein-protein interaction map combined the transcript, protein and phosphoprotein expression changes. Blue represents non-differentially expressed transcripts or proteins. Yellow indicates mRNA or protein expression changes.

Physiological activities are generally accomplished by transient or stable assembled protein complexes; hence, authentication of protein–protein interactions supports profound understanding of cellular process and functions. To elaborate functional networks, we performed clustering of phosphoproteins or proteins and potential partners based on well-established or predicted interactions. Applying this combined protein interaction network (Fig. 4 and Supplementary Table S4), we observed a clustering of different protein complexes. The largest subcluster was composed of structural constituents of ribosomes and proteins involved in translation or ribosomal biogenesis; the majority of proteins in this subcluster increased in the WT compared with *fl*. Other proteins in other clusters were connected through interactions with peroxidases, transcription factors, and kinases. As shown in Fig. 4, tubulin alpha chain-like (Cotton_A_38233) could interact with three proteins in the associated network and directly or indirectly regulate their expression. These proteins were formate-tetrahydrofolate ligase (CotAD_49525), thioredoxin h-type (CotAD_76239). and tubulin beta 8 (EV482881), which were also shown to be key network objects in previous research on cotton fiber initiation^19^.

### Analysis of motifs of differentially expressed phosphoproteins

Phosphorylation sites are footprints of kinase activities. To assess phosphosites conservation, we applied WebLogo to produce sequence logos that were typical within multiple sequence alignments between all phosphosites and differentially expressed ones. Between all phosphosites and differentially expressed ones, the most frequently occurring third amino acid residues were Ser and aspartic acid, respectively (see Supplementary Fig. S4). In total, 9 Ser motifs and 5 Thr motifs were significantly enriched (see Supplementary Table S5). Ten of these motifs were not present in the data from other species (see Supplementary Table S6). The 9 pSer motifs could be grouped into three groups: acidic, basic, and pro-directed. After associating differentially expressed phosphosite motifs with all phosphorylation motifs, the motifs ……S.SD…, …..DS……, and …S..T…… were found to be unique to differentially expressed ones. The motif ……S.SD… is consistent with the classical potential phosphorylation motif SXX(E/D) for casein kinase II phosphorylation, which has been implied in the regulation of cell growth and proliferation^20^. In contrast, the role of …..DS…… and …S..T…… motifs is still unclear.

### Identification of pathways related to fiber initiation by iTRAQ analysis

**Figure 5:**
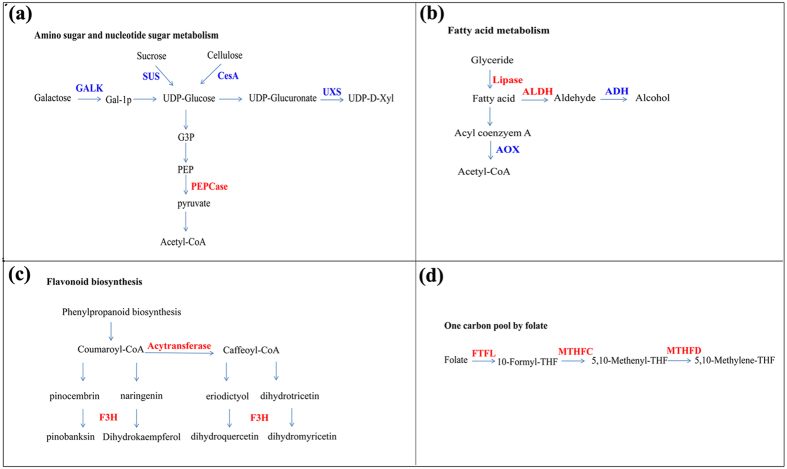
Summary of several biological pathways affected during fiber initiation. Red characters indicate protein up-regulated in WT. Blue characters indicate protein down-regulated in WT. GALK, galactokinase; SUS, sucrose synthase; CesA, cellulose synthase; UXS, UDP-Xyl synthase; PEPCase, Phosphoenol pyruvate carboxylase; MTHFC, methylenetetrahydrofolate cyclohydrolase; MTHFD, methylenetetrahydrofolate dehydrogenase; FTFL, formate-tetrahydrofolate ligase; AOX, acyl-CoA oxidase; ALDH, aldehyde dehydrogenases; ADH, alcohol dehydrogenase; F3H, flavanone 3-hydroxylase.

To obtain further insight into cotton fiber initiation, we constructed a master table summarizing the protein change ratios (see Supplementary Table S1). Some DEPs in the most important pathways are shown in Fig. 5. Many metabolic enzymes were identified, in particular, proteins related to fatty acid metabolism, one-carbon folate metabolism, amino sugar and nucleotide sugar metabolism and flavonoid biosynthesis. Among 10 identified DEPs related to amino sugar and nucleotide sugar metabolism, 9 were down-regulated and 1 was up-regulated in the WT (Fig. 5a). Ten identified proteins were related to fatty acid metabolism, with seven fatty acid biosynthesis-related proteins, including GDSL esterase lipase, lipid transfer protein, and lipid binding-related protein, being abundant in the WT (Fig. 5b). Moreover, five up-regulated proteins identified in the WT—three flavanone 3-hydroxylases and two BAHD acyltransferase—are involved in flavonoid biosynthesis (Fig. 5c). Similarly, three genes involved in one-carbon folate metabolism, namely, methylenetetrahydrofolate dehydrogenase (MTHFD), methylene tetrahydrofolate reductase (MTHFR), and methionine (Met) S-methyltransferase (MMT), had a higher expression level in WT (Fig. 5d). Ethylene, which convers from S-adenosyl-Met, positively affects fiber enlongation growth^21^. One-carbon folate metabolism may affect the ethylene synthesis and impact fiber initiation.

### Enzymatic assays and western blotting

**Figure 6:**
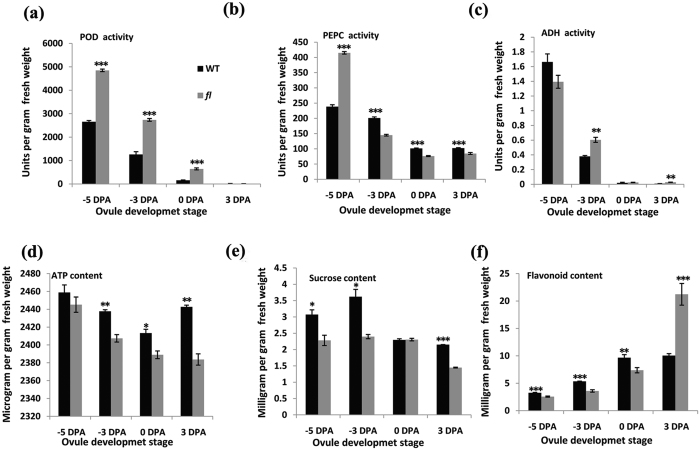
Enzyme activity and metabolite level detection. (**a**) Peroxidases activity. (**b**) Phosphoenolpyruvate carboxylase activity. (**c**) Alcohol dehydrogenase activity. (**d**) Adenosine triphosphate content. (**e**) Sucrose content. (**f**) Flavonoid content. The experiment was repeated three times with similar results. The error bars represent SD (student’s t test, *P < 0.05, **P < 0.01, ***P < 0.001, p = 8.82E-07 at −5 DPA, p = 4.10E-05 at −3 DPA and p = 4.91E-05 at 0 DPA in (**a**), p = 2.03E-06 at −5 DPA, p = 3.15E-05 at −3 DPA, p = 2.72E-05 at 0 DPA and p = 0.00097 at 3 DPA in (**b**), p = 0.006 at −3 DPA and p = 0.002 at 3 DPA in (**c**), p = 0.0044 at −3 DPA, p = 0.026 at 0 DPA and p = 0.0015 at 3 DPA in (**d**), p = 0.0104 at −5 DPA, p = 0.0104 at −3 DPA and p = 9.94E-07 at 3 DPA in (**e**), p = 0.00049 at −5 DPA, p = 0.00016 at −3 DPA, p = 0.0055 at 0 DPA and p = 0.00065 at 3 DPA in (**f**)).

Human serine threonine-protein phosphatase (PPP1CB) was used as an example for the western blotting test. As shown in Supplementary Fig. S5a, actin expression was the same between the WT and *fl*, whereas PPP1CB expression was significantly up-regulated at 0-DPA ovules in WT compared with the mutant. As shown in Supplementary Fig. S5b, glucose-6-phosphate dehydrogenase (G6PDH) activity was up-regulated at 0-DPA ovules in WT compared with those of *fl*. Other enzyme activities and metabolite levels were also detected between the WT and *fl*. Both H_2_O_2_ and reactive oxygen species (ROS) are involved in cotton fiber cell elongation^21^. In the present study, peroxidase activity gradually decreased between −5 and 3 DPA in the WT, with levels in the WT significantly lower than in *fl* (Fig. 6a). This result implies that high peroxidase activity reduces H_2_O_2_ and ROS to affect fiber initiation. Plant cell growth is influenced by cell wall extensibility and cell turgor, with phosphoenolpyruvate carboxylase (PEPC) activity contributing to the extension of fiber cells in cotton^22^. In our study, PEPC activity decreased between −3 and 3 DPA in the WT, with significantly higher activity or content than in *fl* (Fig. 6b). These results were consistent with the protein abundance determined by our iTRAQ quantification. An additional enzyme identified in our data, ethanol-active alcohol dehydrogenase (ADH), exhibited different expression patterns between the WT and *fl*. In particular, ADH abundance was down-regulated in the WT, and the enzymatic assay revealed a similar pattern (Fig. 6c). Furthermore, extracellular ATP depolarizes Arabidopsis root hair membranes and triggers an increase in cytosolic Ca^2+^ 23. In our study, ATP content in the WT was significantly higher than in *fl* between −3 and 3 DPA (Fig. 6d), indicating that ATP is needed for normal cotton fiber initiation and development.

The total fatty acid content of cotton ovules at −3 DPA was characterized by gas chromatography, which revealed that the highest content fatty acids were linoleic acid (C18:2) and palmitic acid (C16:0). WT ovules contained levels of C16:0, stearic acid (C18:0), oleinic acid (C18:1), C18:2, and calendic acid (C18:3) fatty acids higher than those in *fl* ovules (see Supplementary Fig. S6), implying that fatty acids play an important part in fiber development.

The presence of 5 μM dipotassium salt of D-fructose-6-phosphoric acid during *in vitro* culture of cotton ovules can increase the level of fiber initiation in the WT (see Supplementary Fig. S7b). Moreover, application of 10 μM D-glucose (see Supplementary Fig. S7c) can also promote fiber cell initiation. The flavonoid dihydroquercetin exhibited significant effects on fiber development during ovule culture and was able to significantly promote fiber development (see Supplementary Fig. S7d). Sucrose content increased between −5 DPA and −3 DPA in the WT, with levels significantly higher than in the mutant (Fig. 6e). Flavonoid content increased between −5 and 0 DPA in WT ovules, with levels peaking at 0 DPA and significantly higher than in *fl* ovules (Fig. 6f), this result indicates that flavonoids and sucrose participate in fiber initiation.

To confirm the RNA-Seq data, we analyzed nine genes, including genes involved in fatty acid, energy-related, and stress-related metabolism, by quantitative real-time PCR (qRT-PCR) during the two ovule developmental stages. Overall, the 14 qRT-PCR data measurements were significantly correlated with the RNA-Seq results (see Supplementary Table S7, R = 0.702; correlation is significant at the 0.01 level).

### Identification of genomic regions and candidate genes for *li3* and *n2* loci

The *fl* mutant was found to be controlled by two recessive genes, designated *li3* and *n2*. We surveyed 451 F_2_ plants from a self-pollinated segregating population and recorded 324 plants with the normal phenotype (fuzz-linted), 101 plants with the fuzzless-linted phenotype, and 26 plants with the fuzzless-lintless phenotype. These data fitted a 12:3:1 segregation ratio, consistent with the previous study^8^. The dominant *N2* allele (fuzz) is epistatic over the recessive *li3li3* genotype, and *Li3* and *li3li3* display their phenotypes (linted and lintless, respectively) only in the recessive *n2n2* background.

**Figure 7:**
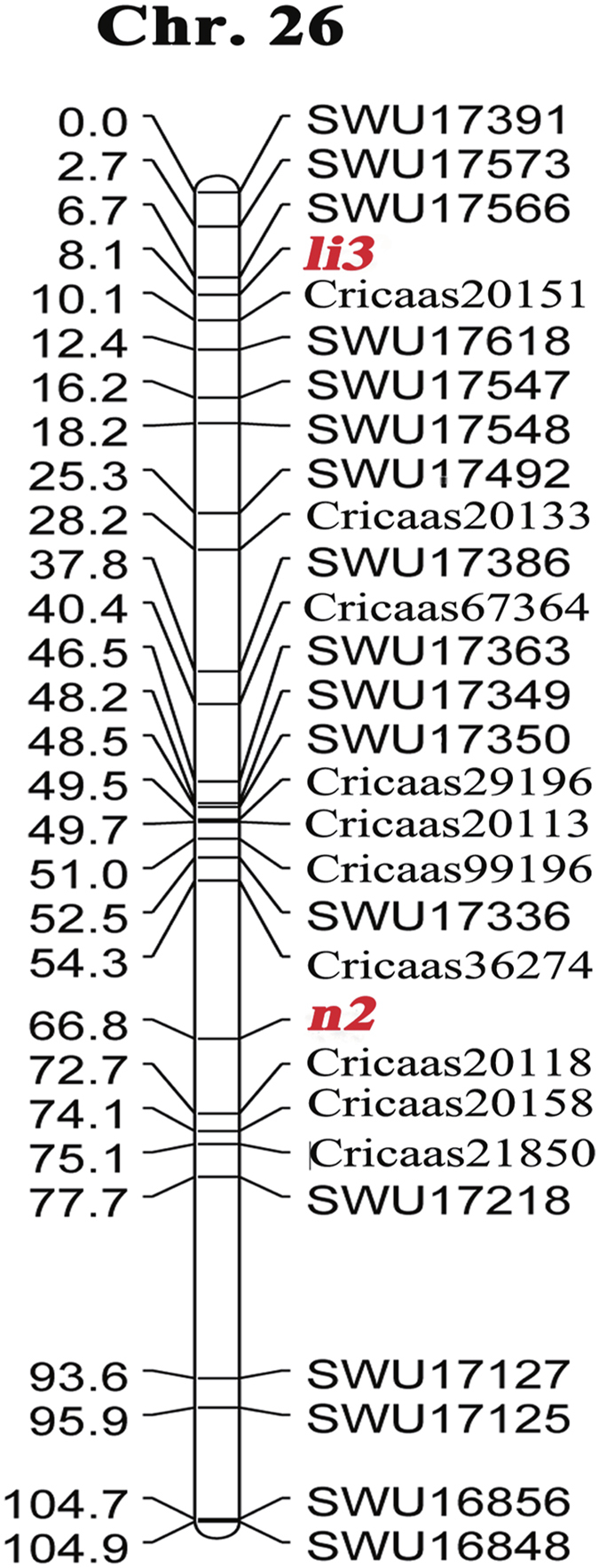
Linkage map of *n2* and *li3* genes.

Of 3,700 simple sequence repeat (SSR) primer pairs, 152 were found to produce polymorphic markers between the WT and *fl*. These informative primers were subsequently used to analyze the 451 F_2_ progeny and their F_1_ hybrid and parents. Single-nucleotide polymorphic (SNP) markers developed in this study were also analyzed using a high-resolution melting curve analysis. Using JoinMap 4.0, *li3* and *n2* were both mapped to chromosome 26 (i.e., D12) using all of the polymorphic SSR markers as well as SNP markers mapped on D12. The *li3* locus was anchored by SWU17566 and Cricaas20151 with a genetic distance of 1.4 and 2 cM, respectively, while *n2* was anchored by Cricaas36274 and Cricaas20158. On the basis of the initial mapping results, additional SSR and SNP markers were developed from the predicted genes in the two regions. Finally, *n2* was located between Cricaas36274 and Cricaas20118 at a genetic distance of 12.5 and 5.9 cM, respectively (Fig. 7). The two genes are distantly located on the same chromosome at a genetic distance of 58.7 cM, with no apparent linkage as shown by the segregation analysis of the two traits (i.e., fuzzless and lintless). Although no closer markers were identified to flank *li3* using the newly developed gene markers, 79 genes including 2 DEGs were identified in the 3.4-cM region flanking *li3*. One DEG (APRR5) was found to contain two SNPs between the WT and *fl*. In the 18.4-cM region flanking the *n2* locus, 301 genes were identified, 9 of which were differentially expressed between the WT and *fl*. SNPs were discovered in only four DEGs: those encoding for a BTB/POZ protein (containing two non-synonymous SNPs), a low molecular-weight heat shock protein (HSP; containing three SNPs- two of which non-synonymous), a glutamate decarboxylase (GAD; containing two non-synonymous), and an uncharacterized protein (containing two non-synonymous SNPs). The DEGs in these two regions were analyzed by qRT-PCR, which yielded results consistent with the RNA-Seq profiling.

## Discussion

Cotton fiber initiation is a faultlessly irreversible, concerted process involving a range of morphological, physiological, and molecular changes that result in the development of a soft fiber with attributes desirable to humans. In an attempt to elucidate the molecular and genetic mechanism of the *fl* mutant, we cataloged differences in mRNA and protein/phosphoprotein abundances by integrated profiling of gene activity using RNA-Seq and iTRAQ technology. This non-gel-based quantitative proteomic method is superior to 2-D gel electrophoresis which tends to identify mostly abundant proteins but suffers from several disadvantages, including difficulty in detecting proteins that are hydrophobic or too large, small, acidic, basic, or scarce^24^. mRNA transcript abundance and cognate protein abundance have long been recognized to be poorly correlated, largely as a consequence of protein degradation and differing translational rates^25^. Our comparison revealed a high consistency between mRNA transcript and protein levels, between transcript and phosphoprotein levels, and between phosphoprotein and protein levels. These data complement the results of previous studies in other species, which have indicated a similar, modest protein/phosphoprotein-transcript or protein–phosphoprotein relationship.

Cotton fiber cell differentiation and initiation is a complicated cell process regulated by diverse pathways and protein interaction networks. The analysis of DEPs or DEGs showed that the WT contained more biological processes compared with *fl*, such as one-carbon folate metabolism and flavonoid biosynthesis. Many genes related to one-carbon folate metabolism were up-regulated in the WT, consistent with previous studies^26^. Other energy-related metabolic pathways were also identified, including amino sugar and nucleotide sugar metabolism and fatty acid metabolism. ROS, including H_2_O_2_ and superoxide anion (O^2−^), and ROS-related genes and proteins were also uncovered in this study. ROS act as secondary messengers in several plant hormone responses at low concentration, while high concentrations of ROS can oxidize lipids and proteins. Appropriate regulation of ROS homeostasis has been suggested to be necessary for cotton fiber initiation^14^. Ascorbate peroxide reduces H_2_O_2_ to water; consequently, the high concentration of ascorbate peroxide in *fl* may affect H_2_O_2_ production, and thereby influence cotton fiber initiation. In the current study, exogenous dihydroquercetin, produced by F_3_H, promoted *in-vitro* fiber initiation development, as did exogenous D-fructose-6-phosphate dipotassium salt and D-galactose. These findings reveal new aspects of cell differentiation and further our understanding of the mechanisms regulating active fiber initiation.

We previously reported the identification of 1,592 phosphosites from 619 phosphoproteins^27^. In the present study, we reanalyzed these phosphosites in light of the completion of sequencing of the upland cotton genome. Aside from nonspecific motifs (e.g., sxxK, sxxR, sD, and sE), analysis of the motifs unique to DEPs revealed three interesting motif classes, including one motif bearing the ……S.SD… signature. The ……S.SD… motif, which is a regulator of cell growth and proliferation, was not observed in other plants or our previous cotton fiber study. We anticipate that the discovery of these novel phosphorylation motifs will provide information about kinase activities and reveal new insights into the cotton fiber differentiation and initiation phosphorylation network.

**Figure 8:**
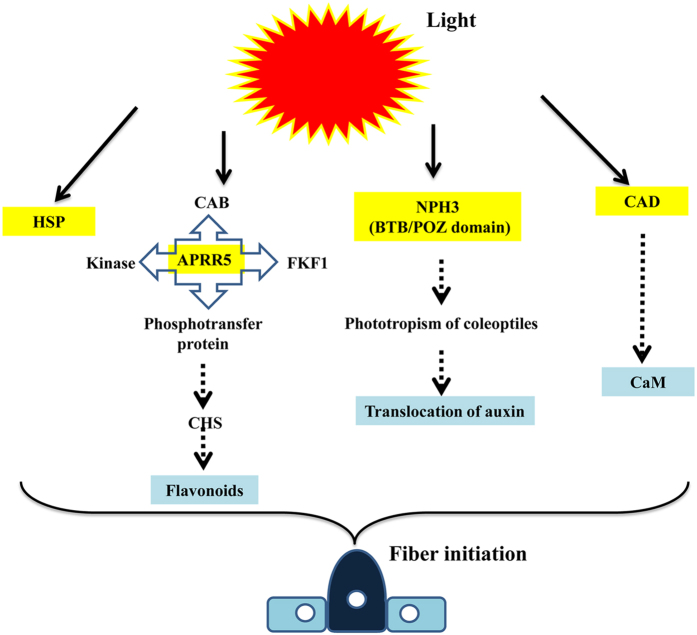
A putative network of five candidate genes regulation on fiber initiation. HSP, heat-shock protein; CAB, timing of CAB expression 1 protein; APRR5, two-component response regulator-like APRR5; FKF1, circadian clock-associated FKF1; CHS, chalcone synthase; NPH3, nonphototropic hypocotyl 3; CAD, glutamate decarboxylase; CaM, calmodulin.

The fiber mutant genes *Li1*, *Li2*, *LiX*, *N1*, and *n2* were previously assigned to chromosomes 22 (D4), 18 (D13), 4 (A4), 12 (A12), and 12 (A12), respectively^5^, but the *li3* gene in the Xuzhou 142 *fl* mutant had never been mapped. The tetraploid cotton genome TM-1 has been recently sequenced^28^. In this study, functional genomic analysis in combination with high resolution mapping of *li3* and *n2* has led to the identification of candidate genes for the *fl* mutant which will facilitate the eventual isolation of the two mutant genes (i.e., *li3* and *n2*). We located *li3* on chromosome 26, where it is anchored by two markers in a 3.4-cM region. The naked seed *n2*, the other studied gene in the *fl* mutant, was previously assigned to chromosome 26 using aneuploids^29^. This assignment was brought into question by a subsequent mapping study, however, as *n2* was not linked to markers on this chromosome, but was instead linked to several markers on its homeologous chromosome 12^9^. Using an F_2_ segregating population, we confirmed that *n2* resides on chromosome 26 in an 18.4-cM region spanned by two markers (Cricaas36274 and Cricaas20118). In addition to our mapping efforts, we applied a transcript profile analysis to mine candidate genes for the *li3* and *n2* locus. Five genes (DEGs with SNPs) in the two regions for *n2* and *li3* were discovered in the current study. The gene encoding a HSP was up-regulated in the WT, whereas the remaining four genes encoding for APRR5, BTB/POZ protein, GAD, and an uncharacterized protein, were down-regulated. The functions of these candidate genes appear to have a relationship with responses to light directly or indirectly, and the network of gene regulation is shown in Fig. 8.

The gene encoding for APRR5 was the only candidate gene (i.e., differentially expressed with SNPs between *fl* and WT) identified in the *li3* region controlling lint fiber initiation. As a light-responsive protein, APRR5 modulates light input to the circadian clock and control of flowering time. Moreover, APRR5 is a negative regulator of shade avoidance response and is also involved in the inhibition of leaf expansion^30^. In addition to interacting with the circadian clock related proteins, APRR5 can be also combined with some kinases and phosphatases, affecting the synthesis of flavonoids. Studies have shown that flavonoids are involved in the process of fiber development^31^. In current study, exogenous flavonoids promoted fiber initiation development by *in-vitro* cotton ovule culture. Cotton fiber development was significantly inhibited under the light conditions *in-vitro*, including fiber initiation rate, ovule surviving rate and fiber length. Considering that the normal growth of cotton fibers occurs in the ovary under the dark conditions, the light-responsive APRR5 protein may participate in cotton fiber development. Although this study has identified a candidate gene ARPP5 for a lint fiber initiation locus for the first time, the mechanism of ARPP5 in regulating lint fiber initiation in cotton remains unknown and needs further studies.

The other four candidate genes encoding for a HSP, a BTB/POZ protein, a GAD, and an uncharacterized protein, are in the *n2* gene region. The synthesis of HSPs due to high temperature exposure, high light intensity, and oxidative stress are a common phenomenon in living organisms^32^. Zhao *et al.*^33^ observed an increase in HSP expression in a cotton WT compared with the *Li1* mutant. They also found that fiber elongation was inhibited after treatment with the HSP90-specific inhibitor radicicol, indicating proper HSP activities were required for fiber elongation. The BTB/POZ domain-containing protein belongs to the phototropic-responsive NPH3 family and is also involved in response to light stimulus. Previous studies have shown that NPH3 is required for phototropism of coleoptiles and lateral translocation of auxin^34^. Auxin plays an important role in fiber initiation. Application of auxin significantly promoted fiber initiation under *in-vitro* cotton ovule culture conditions^35^. The cellular calcium concentration had been found to be increased in response to environmental stimuli including light. As a calmodulin-activated enzyme containing calmodulin-binding domain, GAD is expressed specifically in roots^36^. In a previous research, GhCaM7 promotes cotton fiber elongation by regulating the production of ROS, whereas *GhCaM7* RNA interference inhibits fiber initiation^37^. This observation indicates that Ca^2+^ may modulate early fiber development.

Among the 5 candidate genes, one gene for *li3* encoding a light-responsive APRR5 protein affects the synthesis of flavonoids. The most likely candidate for *n2* is a BTB/POZ protein involved in response to light stimulus and lateral translocation of auxin. Previous studies have shown that flavonoids act as negative regulators of auxin transport^38^. In Arabidopsis, auxin transport is enhanced under the condition of lack of flavonoids (*transparent testa4* mutation [*tt4*]) and reduced with excess flavonoids (*tt7* and *tt3*)^39^. Meanwhile, auxin can regulate flavonol pathway transcripts and induce flavonoid accumulation, altering the relative abundance of kaempferol and quercetin through distinct transcriptional networks^40^. These results may provide a hint that IAA and flavonoid might be viewed as a two-way functional interaction to affect fuzz and lint in cotton. The current study has identified the four candidate genes for the *n2* locus for the first time, although which one or ones are responsible for the *n2* locus is unknown. However, our results have provided a good foundation that will lead to accelerated molecular genetic dissection of the two genes (*n2* and *fl3)* involved in cotton fiber initiation.

## Methods

### Plant materials

Cotton cultivar Xuzhou 142 and its natural isogenic *fl* mutant were provided by the Institute of Cotton Research of CAAS. −3-DPA flower buds and 0-DPA flowers were collected from 60 plants. After dissection, the ovules were frozen in liquid nitrogen (−196 °C) and stored at −80 °C until assayed. Three biological replicates were obtained using 60 plants grown at a uniform growth stage.

### Transcriptome sequencing

Total RNA was extracted as described by John^41^. After removing low-quality reads, we aligned reads to the cotton genome using SOAP2, with up to two mismatches allowed^42^. Gene expression levels were measured as counts of reads per kilobase of exon model per million mapped reads^43^.

### Protein isolation and iTRAQ labeling

Proteins were isolated and purified as described by Pang *et al.*^44^. Total protein was quantified using UV-Vis spectroscopy based on the Bradford method. Samples were digested with 40 μl trypsin buffer at 37 °C for 18 h. iTRAQ reagents were applied to label peptides of proteins according to the manufacturer’s protocol for proteomes (Applied Biosystems, Foster City, CA, USA).

### Phosphopeptide enrichment and MS analysis

TiO_2_ beads were applied to phosphopeptide enrichment. The bound phosphopeptides were washed out with 50 μl NH_4_OH and then vacuum freeze-dried. The MS analysis was performed by HPLC and an Thermo Finnigan (San Jose, CA, USA) according to Griffin *et al.*^45^.

### Database search and bioinformatics

Proteome Discoverer 1.3 and Mascot 2.2 were applied to authenticate proteins and phosphoproteins based on databases combining *G. hirsutum, G. raimondii, G. arboreum*^28^,^46^,^47^ and the CGI (cotton gene index) database. The Blast2GO suite was used to annotate the protein sequences and mine the resulting annotations (i.e., GO and KEGG pathway analyses). Proteins and phosphoproteins that were differentially expressed were used as bait, and their expression changes were mapped onto an interaction network and visualized with Cytoscape 2.8.1^48^. To explore amino acid frequencies around phosphosites, a list of ‘phosphor-13-mers’ were retrieved by applying a BioPerl script. Frequencies of phosphor-13-mer amino acids neighboring the three phosphosites were calculated with the WebLogo program, and their motifs were generated using Motif-X^49^. Correlation coefficients were calculated in JMP (SAS). Alignment analysis of proteins was performed using BioEdit.

### qRT-PCR analysis

Real-time PCR amplifications were performed on a CFX Connect real-time PCR system. The cotton constitutive *histone 3* gene was used as a reference. To detect each sample, three biological replicates were included.

### Western blotting

A total of 10 mg of proteins were separated and transferred onto polyvinylidene difluoride. Filters were blocked for 2 h with blocking buffer and then incubated overnight at 4 °C with monoclonal antibodies raised against PPP1CB (1:1000) or Arabidopsis actin (1:2000). The filters were washed and then incubated with anti-mouse IgG AP-conjugated antibody (1:2000) for 1 h. Protein signals were detected on photographic film.

### Enzymatic metabolite assays

#### Fatty acid analysis

Fatty acids in ovules were extracted and digested as described by Browse *et al.*^50^. Samples were analyzed by gas chromatography (Agilent Technologies, Massy, France) with three biological repeats.

#### ADH assay

As described by Moreno and Pares^51^, ADH catalyzes a reaction involving NAD and ethanol as substrates, with reduced NADH displaying a strong absorption peak at 340 nm. Reaction mixtures for the alcohol dehydrogenase assay contained 3.2% (v/v) ethanol, 0.3 mM sodium phosphate, 7.5 mM β-NAD, 0.003% (w/v) bovine serum albumin, 22 mM sodium pyrophosphate, and 0.1 unit of ADH.

#### G6PD assay

When oxidizing glucose-6-phosphate, G6PD reduces NADP to NADPH. G6PD activity was determined from the change in 340 nm absorbance as described by Tabata *et al.*^52^.

#### PEPC assay

PEPC catalyzes the reaction of HCO_3_^−^ with phosphoenolpyruvate to produce oxalacetate. The decrease in absorbance at 340 nm is directly proportional to the amount of resulting NADH that can be oxidized to NAD^+^ in the presence of malate dehydrogenase. Enzyme activity was determined by monitoring NADH oxidation at 340 nm according to Iglesias and Andreo^53^.

#### Peroxidase assay

Peroxidase activity was determined spectrophotometrically at 470 nm according to its ability to oxidize guaiacol. Reaction mixtures were composed of 0.3 mM H_2_O_2_, 33 mM guaiacol, and 100 mM citric acid/potassium phosphate buffer (pH 5.0). Horseradish peroxidase was used as a standard enzyme.

#### Flavonoid content estimation

Conjugated flavonoid content was measured using acid-hydrolyzed extracts of cotton ovules. Aluminum chloride red complexation was measured colorimetrically at 502 nm^54^.

#### Sucrose content measurement

To measure sucrose content, a differential refractive index detection method was applied. A plain calcium column with an acetonitrile-water (75:25) mobile phase containing 0.01% HPLC amine modifier I was used according to a protocol adapted from Wight and Van Niekerk^55^.

#### ATP content measurement

ATP concentrations in cotton ovules were determined by HPLC. As ATP and its related breakdown products display significant absorbance at 254 nm, peaks were detected and analyzed at that wavelength using a Gold 168 diode array detector.

### Ovule culture and chemical treatment

Cotton −1-DPA ovules were sterilized and cultured in liquid BT medium supplemented with various chemicals containing either 5 μM dipotassium salt of D-fructose 6-phosphorous acid, 10 μM D-glucose, or 50 μM dihydroquercetin. The cultures were incubated at 30 °C in the dark without agitation for 16 h.

### DNA extraction and map construction

DNA was isolated as described by Paterson *et al.*^56^. SSR PCR amplifications and silver staining were conducted as described by Lu *et al.*^57^. JoinMap v4.0 software was used for genetic linkage map construction.

### SNP identification and validation of *li3* and *n2* regions

Using the initial linkage mapping results obtained using SSR markers, we identified genes within the *li3* and *n2* regions based on the sequenced upland cotton genome. DEGs were subsequently identified between the WT and *fl* for SNP analysis. Primer pairs were designed for DEGs with SNPs to perform PCR and high-resolution melting analysis. New markers developed using this positional candidate gene approach were added to the linkage map.

## Additional Information

**How to cite this article**: Ma, Q.-F. *et al.* Integrative transcriptome, proteome, phosphoproteome and genetic mapping reveals new aspects in a fiberless mutant of cotton. *Sci. Rep.* **6**, 24485; doi: 10.1038/srep24485 (2016).

## Supplementary Material

Supplementary Information

Supplementary Table S1

Supplementary Table S2

Supplementary Table S3

Supplementary Table S4
