# Supplementary material for: Integrative transcriptome, proteome, phosphoproteome and genetic mapping reveals new aspects in a fiberless mutant of cotton
Source: Sci Rep. 2016 Apr 14;6:24485. doi: 10.1038/srep24485 (PMC4830928; doi:10.1038/srep24485)
Supplement: Supplementary Information [file srep24485-s1.pdf]

Supplementary Information

Integrative transcriptome, proteome, phosphoproteome and genetic mapping reveals new aspects in a fiberless mutant of cotton

Qi-Feng Ma[1], Chun-Hui Wu[1], Man Wu[1], Wen-Feng Pei[1], Xing-Li Li[1], Wen-Kui Wang[1], Jinfa Zhang[2,*], Ji-Wen Yu[1,*], and Shu-Xun Yu[1,*]

[1] State Key Laboratory of Cotton Biology, Institute of Cotton Research of CAAS, Anyang, Henan 455000, China

[2] Department of Plant and Environmental Sciences, New Mexico State University, Las Cruces, NM 88003, USA

* To whom correspondence should be addressed: No. 38, Institute of Cotton Research of CAAS, Huanghe Rd., Anyang, Henan 455000, P.R. China, Tel: +86-372-2562275, Fax: +86-372-2562201, Email: ysx195311@163.com

Supplementary figure and table legends

Supplementary Fig. S1. The experimental workflow in this study. WT −3 DPA and WT 0 DPA: −3-DPA and 0-DPA ovules from WT. *fl* −3 DPA and *fl* 0 DPA: −3-DPA and 0-DPA ovules from *fl* mutant. Bars: 200 μm in WT −3 DPA and *fl* −3 DPA; 20 μm in WT 0 DPA and *fl* 0 DPA.

Supplementary Fig. S2. Alternative splicing events in the common genes between DEGs and DEPs. Yellow bar illustrated the gene sequences same as the cotton genome annotated genes. Blue bar indicated sequences different from the annotated genes in the WT. Red bar indicated sequences different from the annotated genes in the *fl*.

Supplementary Fig. S3. comparison of phosphosites between CotAD_44282 and homologs in other species.

Supplementary Fig. S4. Phosphoraylation motifs between all phosphosites (a) and differential phosphosites (c). (b, d) Top five phosphoraylation motifs.

Supplementary Fig. S5. Western blotting and enzymatic assay validation of selected differentially expressed proteins. (a) Western blotting of actin and PPP1CB expression between WT and *fl* at 0 DPA. (b) Enzymatic assay analysis of the G6PDH enzyme. Three biological replicates were performed. The error bars represent SD (student's t test, ***$p < 0.001$, $p = 5.34E-05$ at -5 DPA stage, $p = 0.00057$ at 0 DPA stage, $p = 5.87E-05$ at 3 DPA stage).

Supplementary Fig. S6. Quantification of fatty acids of WT and *fl* ovules at -3 DPA. (student's t test, *$P < 0.05$, **$P < 0.01$, $p = 0.011$ at C16:0, $p = 0.031$ at C18:0, p =

0.0028 at C18:1, $p = 0.0015$ at C18:2, $p = 0.0015$ at C18:3).

Supplementary Fig. S7. Cotton ovule culture in vitro. Scanning electron of unfertilized ovule surface treated with (a) none, (b) 5 μM D - fructose di-*potassium salts* of 6-*phosphorous acid, (c)* 10 μM *D-glucose*, (d) 50 μM dihydroquercetin. Bars = 100 μm.

Supplementary Table S1. The differentially expressed genes (sheet 1-4), proteins (sheet 5-8), and phosphoproteins (sheet 9-12) list. A, Xuzhou 142 WT -3 DPA ovules; B, Xuzhou 142 WT 0 DPA ovules; C, Xuzhou 142 *fl* -3 DPA ovules; D, Xuzhou 142 *fl* 0 DPA ovules.

Supplementary Table S2. A complete list of detected genes (sheet 1), proteins (sheet 2), and phosphoproteins (sheet 3).

Supplementary Table S3. The previously reported genes, proteins and phosphoproteins.

Supplementary Table S4. Protein-protein interaction network.

Supplementary Table S5. Phosphorylation motifs distribution between the phosphorylated protein (sheet 1-2) and the differentially phosphorylated protein (sheet 3-4).

Supplementary Table S6. Differentially phosphorylated protein motifs identified in this study.

Supplementary Table S7. The primer sequences list and gene expression profiles following RNA-Seq and qRT-PCR.

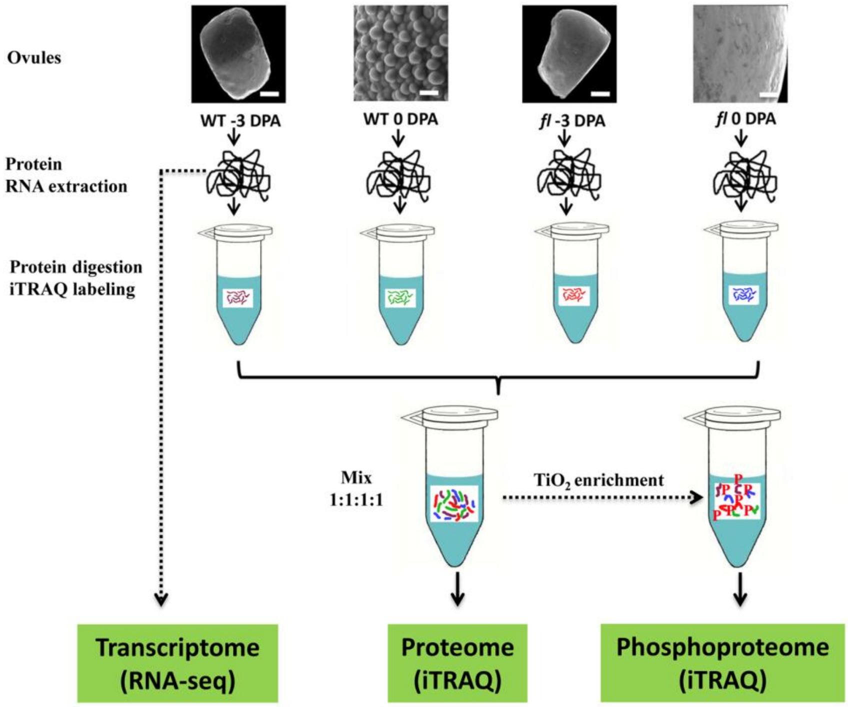

Supplementary Fig. S1. The experimental workflow in this study. WT −3 DPA and WT 0 DPA: −3-DPA and 0-DPA ovules from WT. *fl* −3 DPA and *fl* 0 DPA: −3-DPA and 0-DPA ovules from *fl* mutant. Bars: 200 μm in WT −3 DPA and *fl* −3 DPA; 20 μm in WT 0 DPA and *fl* 0 DPA.

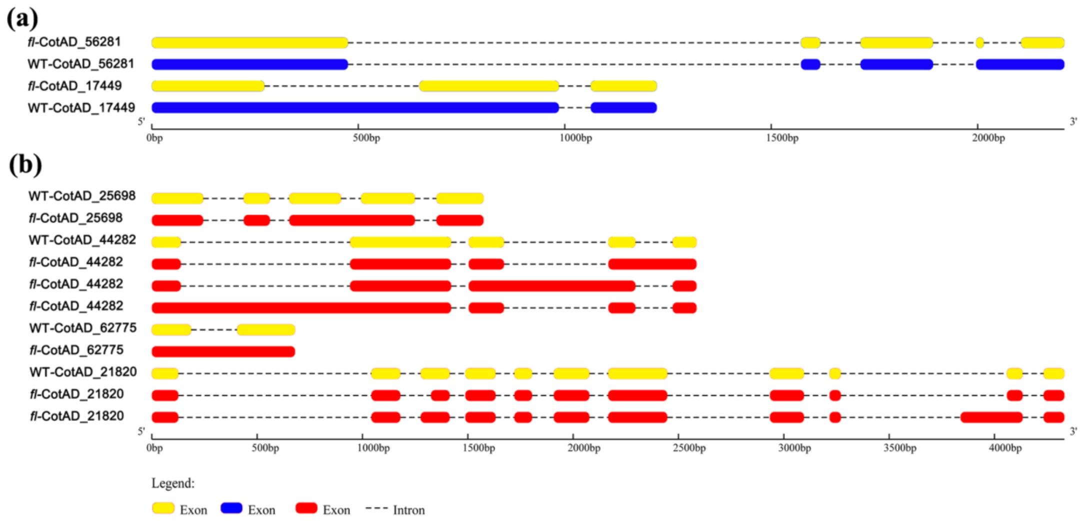

Supplementary Fig. S2. Alternative splicing events in the common genes between DEGs and DEPs. Yellow bar illustrated the gene sequences same as the cotton genome annotated genes. Blue bar indicated sequences different from the annotated genes in the WT. Red bar indicated sequences different from the annotated genes in the *fl*.

```
CotAD_44282  1    -MLMSRALSRVSKRVGARSLLL---SAPKSFQSPSLSTQFHSLVSESPNKVITNDVCLLQNSNLHWNAFRRFGISSSASP
P3DB-13491   1    -----------MEFLSTSMLAA---EAEESYRSGHNSTHLFP--------VQVNFLCPSINS-----STSKFGFSSSASV
P3DB-5227    1    MAAAARLLARISRQGVASAAAARRQAEAAALLGASAGRHLAPPCSSIKALPLLNQPRLYSTS-----TFQRFGFSSSAP-

CotAD_44282  77   EPSEKEHGSAVDN---NGQDPVKPSED----TIPGEAEATKESG-FNSESQNAMPQSDKRRRKAAKRTAFSDSDSESDGD
P3DB-13491   54   ETEGKE-GNNVHNGNFTSADPAKTSEEAEEIDQPGQAKSADQTEESGSISADSHSQTVKRRRRSIKRTAFSDSDSDSESD
P3DB-5227    75   QQDDKAANKQTEDGVNKSTQSEASNETN---SSPG-TENASQAG-----SQDSVPQS-NRRRRGTKRTAFSDSDTE-DLD

CotAD_44282  149  LSMADLVKLVEEKEELLKTKQTEIEQMKDKVVRTLAEMENVMARTRREAENSKKFAIQNFVKGLLDVADNLGRASTHVKG
P3DB-13491   133  LSRDDLIKLVAEKEELLKLKKKEIEKMQDKVLRTYAEMENVMDRTRREAENSRKFAIQNFAKSLLDVADNLGRASSVVKE
P3DB-5227    144  LSKEDLTKLVLEKEELLKSKDEEVKDMKDKVLRSYAEMENVIARTKRESDNAKKYAVQGFSKSLLDVADNLSRASSVVKE

CotAD_44282  229  SFSKIDESND-TAGAVPLLKTLLEGVEMTEKQLGEVFRKFGVEKFDPTNEPFDPHRHNAVFQVPDNSKPPGTVAHVLKAG
P3DB-13491   213  SFSKIDTSSDSSAEAVKLLKTLLEGVEMTEKQLSEALKKFGVEKFDPTNEPFDPHRHNAVFKVPDGSKPPGTVAAVLKAG
P3DB-5227    224  SFSKIDTSNE-SAEAVKLLNTLLEGVEMTEKQLGEVFKKFGVEKFDPLNEKFDPNKHAALFQIPDPSKPSGTVAAVVKVG

CotAD_44282  308  YMLYDRVIRPAEVGVTQATDDGATENNTGDKVSDA
P3DB-13491   293  YLLHDRVIRPAEVGVTQAKENGNSAE---------
P3DB-5227    303  YMLHDRVLRPAEVGVTEGGPIEEEPEEKSDKSD--
```

Supplementary Fig. S3. comparison of phosphosites between CotAD_44282 and homologs in other species.

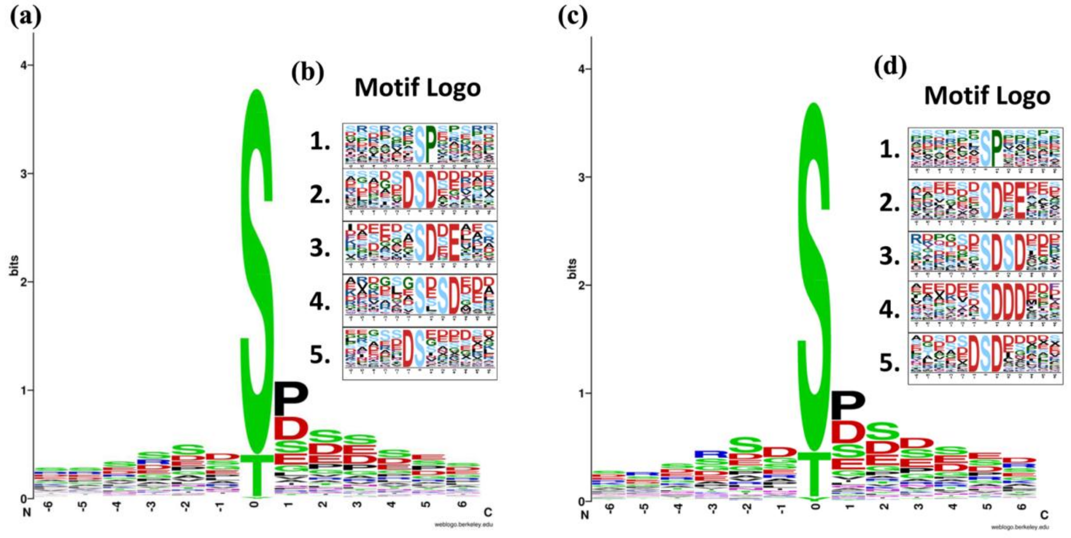

Supplementary Fig. S4. Phosphoraylation motifs between all phosphosites (a) and differential phosphosites (c). (b, d) Top five phosphoraylation motifs.

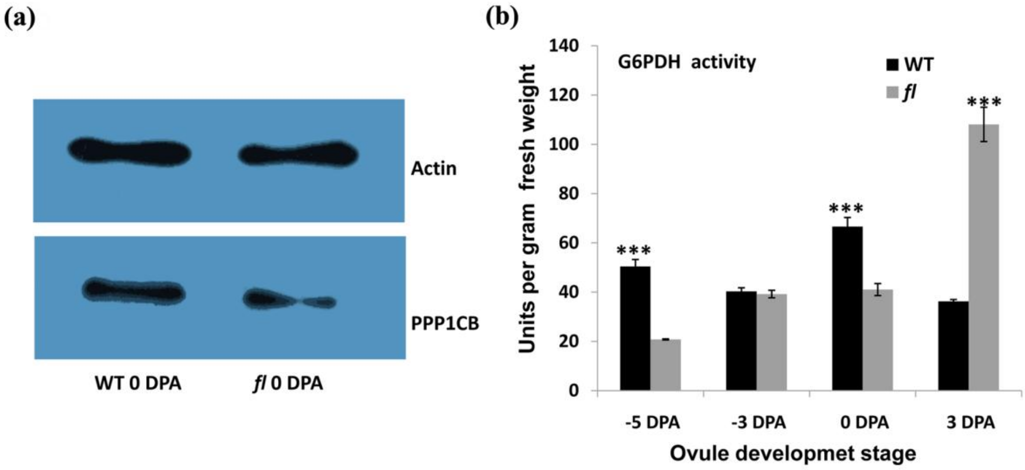

Supplementary Fig. S5. Western blotting and enzymatic assay validation of selected differentially expressed proteins. (a) Western blotting of actin and PPP1CB expression between WT and *fl* at 0 DPA. (b) Enzymatic assay analysis of the G6PDH enzyme. Three biological replicates were performed. The error bars represent SD (student's t test, ***$p < 0.001$, $p = 5.34E-05$ at -5 DPA stage, $p = 0.00057$ at 0 DPA stage, $p = 5.87E-05$ at 3 DPA stage).

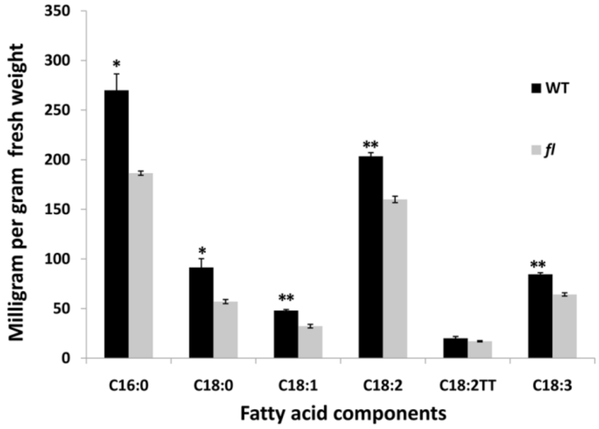

Supplementary Fig. S6. Quantification of fatty acids of WT and *fl* ovules at -3 DPA. (student's t test, *P < 0.05, **P < 0.01, p = 0.011 at C16:0, p = 0.031 at C18:0, p = 0.0028 at C18:1, p = 0.0015 at C18:2, p = 0.0015 at C18:3).

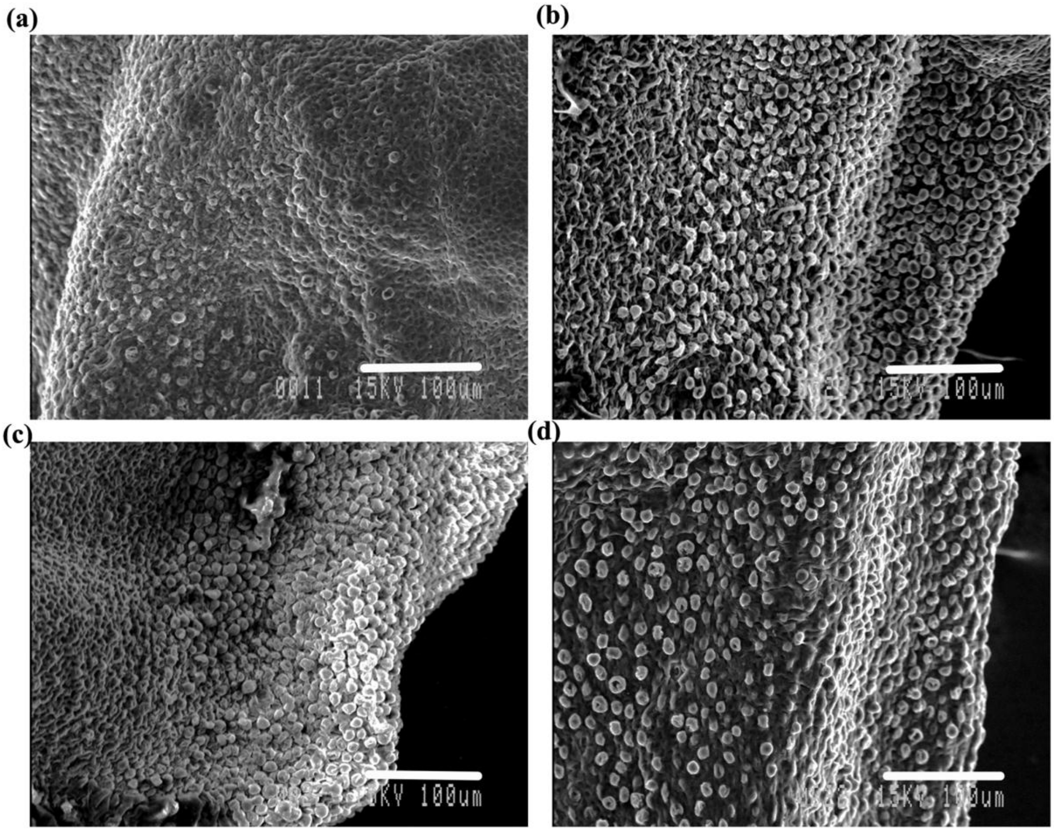

Supplementary Fig. S7. Cotton ovule culture in vitro. Scanning electron of unfertilized ovule surface treated with (a) none, (b) 5 μM D - fructose di-*potassium salts* of 6-*phosphorous acid, (c)* 10 μM *D-glucose*, (d) 50 μM dihydroquercetin. Bars = 100 μm.

Supplementary Table S3. The previously reported genes, proteins and phosphoproteins.

Gene list	Type
CotAD_00199	The previously reported gene
CotAD_00236	The previously reported gene
CotAD_00696	The previously reported gene
CotAD_00940	The previously reported gene
CotAD_01748	The previously reported gene
CotAD_02255	The previously reported gene
CotAD_02378	The previously reported gene
CotAD_02433	The previously reported gene
CotAD_02553	The previously reported gene
CotAD_02961	The previously reported gene
CotAD_03497	The previously reported gene
CotAD_04193	The previously reported gene
CotAD_04477	The previously reported gene
CotAD_04628	The previously reported gene
CotAD_04637	The previously reported gene
CotAD_05386	The previously reported gene
CotAD_05977	The previously reported gene
CotAD_06350	The previously reported gene
CotAD_06406	The previously reported gene
CotAD_06986	The previously reported gene
CotAD_07026	The previously reported gene
CotAD_07129	The previously reported gene
CotAD_07491	The previously reported gene
CotAD_07567	The previously reported gene
CotAD_09649	The previously reported gene
CotAD_10182	The previously reported gene
CotAD_10228	The previously reported gene
CotAD_10611	The previously reported gene
CotAD_10936	The previously reported gene
CotAD_11005	The previously reported gene
CotAD_11036	The previously reported gene
CotAD_12715	The previously reported gene
CotAD_12781	The previously reported gene
CotAD_12926	The previously reported gene
CotAD_13206	The previously reported gene
CotAD_13625	The previously reported gene
CotAD_13946	The previously reported gene
CotAD_14462	The previously reported gene
CotAD_14663	The previously reported gene

CotAD_14769	The previously reported gene
CotAD_14780	The previously reported gene
CotAD_15105	The previously reported gene
CotAD_15396	The previously reported gene
CotAD_15507	The previously reported gene
CotAD_15554	The previously reported gene
CotAD_15724	The previously reported gene
CotAD_16059	The previously reported gene
CotAD_16314	The previously reported gene
CotAD_16520	The previously reported gene
CotAD_16680	The previously reported gene
CotAD_16945	The previously reported gene
CotAD_17026	The previously reported gene
CotAD_17473	The previously reported gene
CotAD_17915	The previously reported gene
CotAD_18107	The previously reported gene
CotAD_18860	The previously reported gene
CotAD_19011	The previously reported gene
CotAD_19158	The previously reported gene
CotAD_19231	The previously reported gene
CotAD_19591	The previously reported gene
CotAD_20153	The previously reported gene
CotAD_20567	The previously reported gene
CotAD_21208	The previously reported gene
CotAD_21924	The previously reported gene
CotAD_22244	The previously reported gene
CotAD_22834	The previously reported gene
CotAD_23077	The previously reported gene
CotAD_23731	The previously reported gene
CotAD_24107	The previously reported gene
CotAD_24297	The previously reported gene
CotAD_24572	The previously reported gene
CotAD_25082	The previously reported gene
CotAD_25094	The previously reported gene
CotAD_25416	The previously reported gene
CotAD_25559	The previously reported gene
CotAD_25698	The previously reported gene
CotAD_26597	The previously reported gene
CotAD_27919	The previously reported gene
CotAD_27924	The previously reported gene
CotAD_28784	The previously reported gene
CotAD_29214	The previously reported gene
CotAD_29325	The previously reported gene
CotAD_30021	The previously reported gene

CotAD_30233	The previously reported gene
CotAD_30242	The previously reported gene
CotAD_30567	The previously reported gene
CotAD_31283	The previously reported gene
CotAD_31379	The previously reported gene
CotAD_31489	The previously reported gene
CotAD_31689	The previously reported gene
CotAD_32299	The previously reported gene
CotAD_32615	The previously reported gene
CotAD_32773	The previously reported gene
CotAD_32973	The previously reported gene
CotAD_34067	The previously reported gene
CotAD_35876	The previously reported gene
CotAD_36532	The previously reported gene
CotAD_36594	The previously reported gene
CotAD_36667	The previously reported gene
CotAD_36879	The previously reported gene
CotAD_37838	The previously reported gene
CotAD_38070	The previously reported gene
CotAD_38156	The previously reported gene
CotAD_38430	The previously reported gene
CotAD_38656	The previously reported gene
CotAD_40115	The previously reported gene
CotAD_40215	The previously reported gene
CotAD_40334	The previously reported gene
CotAD_40395	The previously reported gene
CotAD_41089	The previously reported gene
CotAD_41275	The previously reported gene
CotAD_41643	The previously reported gene
CotAD_41989	The previously reported gene
CotAD_42684	The previously reported gene
CotAD_42828	The previously reported gene
CotAD_43297	The previously reported gene
CotAD_43445	The previously reported gene
CotAD_44282	The previously reported gene
CotAD_44359	The previously reported gene
CotAD_46044	The previously reported gene
CotAD_46568	The previously reported gene
CotAD_46764	The previously reported gene
CotAD_47132	The previously reported gene
CotAD_47921	The previously reported gene
CotAD_48194	The previously reported gene
CotAD_48277	The previously reported gene
CotAD_48384	The previously reported gene

CotAD_50040	The previously reported gene
CotAD_50241	The previously reported gene
CotAD_51014	The previously reported gene
CotAD_52389	The previously reported gene
CotAD_52859	The previously reported gene
CotAD_53319	The previously reported gene
CotAD_53565	The previously reported gene
CotAD_53608	The previously reported gene
CotAD_53880	The previously reported gene
CotAD_53983	The previously reported gene
CotAD_54337	The previously reported gene
CotAD_54350	The previously reported gene
CotAD_54841	The previously reported gene
CotAD_54934	The previously reported gene
CotAD_56911	The previously reported gene
CotAD_57769	The previously reported gene
CotAD_58038	The previously reported gene
CotAD_58064	The previously reported gene
CotAD_58358	The previously reported gene
CotAD_58749	The previously reported gene
CotAD_59222	The previously reported gene
CotAD_60054	The previously reported gene
CotAD_60970	The previously reported gene
CotAD_61466	The previously reported gene
CotAD_62037	The previously reported gene
CotAD_62802	The previously reported gene
CotAD_64148	The previously reported gene
CotAD_64256	The previously reported gene
CotAD_64448	The previously reported gene
CotAD_66378	The previously reported gene
CotAD_66643	The previously reported gene
CotAD_67194	The previously reported gene
CotAD_67601	The previously reported gene
CotAD_68165	The previously reported gene
CotAD_68168	The previously reported gene
CotAD_68363	The previously reported gene
CotAD_68885	The previously reported gene
CotAD_69714	The previously reported gene
CotAD_70161	The previously reported gene
CotAD_70244	The previously reported gene
CotAD_71359	The previously reported gene
CotAD_71648	The previously reported gene
CotAD_72400	The previously reported gene
CotAD_73030	The previously reported gene

CotAD_73221	The previously reported gene
CotAD_73692	The previously reported gene
CotAD_74529	The previously reported gene
CotAD_74851	The previously reported gene
CotAD_74952	The previously reported gene
CotAD_74978	The previously reported gene
CotAD_75003	The previously reported gene
CotAD_75313	The previously reported gene
CotAD_75563	The previously reported gene
CotAD_76113	The previously reported gene
CotAD_76165	The previously reported gene
CotAD_76654	The previously reported gene
CotAD_76868	The previously reported gene
CotAD_49525	The previously reported protein
Cotton_D_gene_10000638	The previously reported protein
CotAD_11036	The previously reported protein
CotAD_07844	The previously reported protein
CotAD_24342	The previously reported protein
TC251823	The previously reported protein
Cotton_D_gene_10017453	The previously reported protein
CotAD_42608	The previously reported protein
CotAD_51639	The previously reported protein
Cotton_D_gene_10017826	The previously reported protein
CotAD_21698	The previously reported protein
Cotton_A_11375	The previously reported protein
CotAD_09528	The previously reported protein
Cotton_D_gene_10008766	The previously reported protein
EV482881	The previously reported protein
CotAD_05159	The previously reported phosphoprotein
CotAD_07377	The previously reported phosphoprotein
CotAD_07810	The previously reported phosphoprotein
CotAD_09667	The previously reported phosphoprotein
CotAD_14342	The previously reported phosphoprotein
CotAD_24069	The previously reported phosphoprotein
CotAD_27572	The previously reported phosphoprotein
CotAD_31803	The previously reported phosphoprotein
CotAD_39741	The previously reported phosphoprotein
CotAD_40275	The previously reported phosphoprotein
CotAD_44190	The previously reported phosphoprotein
CotAD_47595	The previously reported phosphoprotein
CotAD_54943	The previously reported phosphoprotein
CotAD_60172	The previously reported phosphoprotein
CotAD_61595	The previously reported phosphoprotein
CotAD_64492	The previously reported phosphoprotein

CotAD_65328	The previously reported phosphoprotein
CotAD_66948	The previously reported phosphoprotein
CotAD_72377	The previously reported phosphoprotein
Cotton_A_01216	The previously reported phosphoprotein
Cotton_A_01339	The previously reported phosphoprotein
Cotton_A_03692	The previously reported phosphoprotein
Cotton_A_06948	The previously reported phosphoprotein
Cotton_A_07472	The previously reported phosphoprotein
Cotton_A_19569	The previously reported phosphoprotein
Cotton_A_25396	The previously reported phosphoprotein
Cotton_A_29925	The previously reported phosphoprotein
Cotton_A_38233	The previously reported phosphoprotein
Cotton_D_gene_10007707	The previously reported phosphoprotein
Cotton_D_gene_10014636	The previously reported phosphoprotein
Cotton_D_gene_10018388	The previously reported phosphoprotein
Cotton_D_gene_10026593	The previously reported phosphoprotein
Cotton_D_gene_10027878	The previously reported phosphoprotein
Cotton_D_gene_10028753	The previously reported phosphoprotein
Cotton_D_gene_10035684	The previously reported phosphoprotein
Cotton_D_gene_10040894	The previously reported phosphoprotein
TC229918	The previously reported phosphoprotein
Cotton_A_19478	The previously reported phosphoprotein
Cotton_A_03590	The previously reported phosphoprotein
CotAD_12923	The previously reported phosphoprotein
TC247099	The previously reported phosphoprotein
CotAD_44734	The previously reported phosphoprotein
TC271223	The previously reported phosphoprotein
CotAD_52648	The previously reported phosphoprotein
CotAD_74126	The previously reported phosphoprotein
Cotton_A_12625	The previously reported phosphoprotein
CotAD_38392	The previously reported phosphoprotein
CotAD_51219	The previously reported phosphoprotein
CotAD_18320	The previously reported phosphoprotein
CotAD_40997	The previously reported phosphoprotein
TC233322	The previously reported phosphoprotein
CotAD_50655	The previously reported phosphoprotein

Supplementary Table S6. Differentially phosphorylated protein motifs identified in this study.

NO	Motif	Special for DEPs or not	Found in other species	Motif pattern	Motif category
1	......SP.....	No	Yes	sP	Pro-directed
2	.....DSD.....	No	Yes	[D/E]s	Acidic
3	......SD.E...	No	Yes	sXX[D/E]	Acidic
4	......S.SD...	Special	Novel	sXX[D/E]	Acidic
5	.....DS......	Special	Yes	[D/E]s	Acidic
6	......S.D....	No	Yes	sX[D/E]	Acidic
7	......SD.....	No	Novel	s[D/E]	Acidic
8	...R..S......	No	Yes	RXXs	Basic
9	......SE.....	No	Yes	s[D/E]	Acidic
10	......TP...P.	No	Novel	Unclear	Unclear
11	......TP.....	No	Yes	Unclear	Unclear
12	......TD.....	No	Novel	Unclear	Unclear
13	......TSD....	No	Yes	Unclear	Unclear
14	...S..T......	Special	Yes	Unclear	Unclear

Supplementary Table S7. The primer sequences list and gene expression profiles following RNA-Seq and qRT-PCR.

Gene list	Forward	Reverse	RNA-Seq(Expression)		qRT-PCR (Expression)	
			**-3_WT:-3_*fl***	**0_WT:0_*fl***	**-3_WT:-3_*fl***	**0_WT:0_*fl***
CotAD_35784	AGACTCGCCATTGCTGCTC	ATAATCCGGCCACAAACCA	3.05	4.2	2.5	9.65
CotAD_60970	ACTTGCCTTACGGGATTGA	CCAGTTGCTGTCCGGTCTC	8.3	9.3	3.5	6.87
CotAD_68165	CATCAGTGGAGCAAAGGGTC	GGTAGGAGCAATGGCAGGA	2.01	2.3	2.6	6.72
CotAD_13206	GGTATTCGGACACGTTAATG	AAGACAGAAGAACCAGATGG		4.17		5.5
CotAD_52285	ATAGCGAAACCGACTACCC	GCTGAGCAACGGCACATAA		2.38		4.26
CotAD_07889	GACCCAACAGTTCCCAACC	GGACACCTTCCCATCTTTC	1.37	1.22	1.67	4
CotAD_42040	TAACCCGAGTTTACGAGGAT	AGGCGAGTGAAGTTCTACCAT	-3.1	-2.69	0.25	0.429
CotAD_12714	TTGCTGCTCAATTCTTGGA	AGGACTTGCTTCACTAGGA	-2.6		0.12	
CotAD_22057	GCAGGATGTTGAAGATGG	CCCAATTAAGAGAAAGAGGA	-1.59		0.58	
